# Expression of classic cadherins and δ-protocadherins in the developing ferret retina

**DOI:** 10.1186/1471-2202-10-153

**Published:** 2009-12-22

**Authors:** Johannes Etzrodt, K Krishna-K, Christoph Redies

**Affiliations:** 1 Institute of Anatomy I, University of Jena School of Medicine, Teichgraben 7, D-07743 Jena, Germany

## Abstract

**Background:**

Cadherins are a superfamily of calcium-dependent adhesion molecules that play multiple roles in morphogenesis, including proliferation, migration, differentiation and cell-cell recognition. The subgroups of classic cadherins and δ-protocadherins are involved in processes of neural development, such as neurite outgrowth, pathfinding, target recognition, synaptogenesis as well as synaptic plasticity. We mapped the expression of 7 classic cadherins (CDH4, CDH6, CDH7, CDH8, CDH11, CDH14, CDH20) and 8 δ-protocadherins (PCDH1, PCDH7, PCDH8, PCDH9, PCDH10, PCDH11, PCDH17, PCDH18) at representative stages of retinal development and in the mature retina of the ferret by in situ hybridization.

**Results:**

All cadherins investigated by us are expressed differentially by restricted populations of retinal cells during specific periods of the ferret retinogenesis. For example, during embryonic development, some cadherins are exclusively expressed in the outer, proliferative zone of the neuroblast layer, whereas other cadherins mark the prospective ganglion cell layer or cells in the prospective inner nuclear layer. These expression patterns anticipate histogenetic changes that become visible in Nissl or nuclear stainings at later stages. In parallel to the ongoing development of retinal circuits, cadherin expression becomes restricted to specific subpopulations of retinal cell types, especially of ganglion cells, which express most of the investigated cadherins until adulthood. A comparison to previous results in chicken and mouse reveals overall conserved expression patterns of some cadherins but also species differences.

**Conclusions:**

The spatiotemporally restricted expression patterns of 7 classic cadherins and 8 δ-protocadherins indicate that cadherins provide a combinatorial adhesive code that specifies developing retinal cell populations and intraretinal as well as retinofugal neural circuits in the developing ferret retina.

## Background

The vertebrate retina develops as a ventral outgrowth of the forebrain vesicle; invagination of the primary optic vesicle leads to a two-layered optic cup that differentiates into an outer, pigmented epithelium and an inner, multilayered, neural epithelium, which is the focus of our study. The mature neural retina comprises distinct sets of neurons, each with a characteristic morphology, location and connectivity. Together, they form a highly sophisticated network, arranged in distinct layers [reviewed in [1,2]]. Retinal development involves the processes of cellular proliferation, migration and differentiation. These processes are mediated by molecular mechanisms similar to those in the rest of the brain.

Many studies have investigated the organization and developmental dynamics of the mammalian retina in recent years [reviewed in [3]] and some of the molecular events that regulate retinal neuronal development have been elucidated. For example, genes that play a role in retinogenesis include gene regulatory factors like the homeobox transcription factor Pax6 or the transcription factors MASH-1 and NeuroD, surface proteins like Ng-CAM or N-cadherin (cadherin-2), the Notch-Delta signaling pathway, diffusable factors like retinoic acid, sonic hedgehog or NGF, extracellular matrix molecules like β1-integrins and synaptic adhesion molecules like sidekick, synaptophysin and synaptotagmin [reviewed in [4-6]].

To identify additional potential morphoregulatory molecules as well as structural markers for developmental events, we focus on cadherins in the present study. Cadherins are Ca^2+^-dependent cell surface glycoproteins, which mediate cell adhesion and also have functions in cell signaling from early stages of animal evolution [reviewed in [7]]. The more than 100 members of the cadherin superfamily in vertebrates are grouped into several subfamilies designated as classic, desmosomal, Flamingo/CELSR, Fat-type cadherins, protocadherins and others [reviewed in [8]]. The well-studied classic cadherins mediate strong cell-cell interactions and prefer homophilic over heterophilic binding. They constitute integral components of adherens junctions, interact with the cytoskeleton through catenins and participate in many signaling pathways. Most members of this subfamily are expressed in the nervous system and were proposed to be involved in the functional regionalization of the brain and in the formation, maintenance and plasticity of neural circuits [reviewed in [9-11]]. Protocadherins constitute the largest cadherin subfamily and contain several subgroups, such as α-, β- and δ-protocadherins; they seem to have arisen with the onset of vertebrates [7]. Protocadherins exhibit weaker binding activity than classic cadherins but have a high potential for intracellular signaling and play crucial roles in neuronal development although their precise functions are largely unknown [12-14]. A more recently discovered subgroup are the non-clustered δ-protocadherins [15,16]. The differential and restricted expression patterns of individual cadherins in specific brain regions and neuronal subpopulations suggests that classic cadherins as well as δ-protocadherins provide an adhesive code for developing and mature neural circuits of the central nervous system [reviewed in [9,17]].

Loss-of-function studies demonstrate that classic cadherins and δ-protocadherins play a crucial role in vertebrate retinogenesis. They have multiple morphoregulatory functions in retinal proliferation, migration, differentiation and layer formation as well as in axonal outgrowth, pathfinding, target recognition and synaptogenesis. In general, cadherin-deficient eyes have small, dislayered and disconnected retinae [reviewed in [10,11]]. However, not only the establishment of intraretinal neural networks and retinofugal projections, but also synaptic plasticity [18] and transport processes in photoreceptors [19,20] in the adult retina are found to be cadherin-regulated. In retinal pathology, Usher syndrome type 1 [19], different syndromes with macular dystrophy in humans [21] and retinoblastoma [22,23] are associated with cadherin dysfunction.

In the avian and mammalian retina, the developmental expression patterns of several classic cadherins and δ-protocadherins have been mapped, including, in chicken, cadherin-2 (CDH2), CDH3, CDH4, CDH6B, CDH7 [24] and protocadherin-10 (PCDH10) [25]. In the developing mouse retina, Faulkner-Jones et al. [26] identified several cadherins (CDH1, CDH2, CDH3, CDH4, CDH5, CDH6, CDH10, CDH11, CDH12, CDH14) by immunoblotting and examined the expression of "CDH7", which actually corresponds to CDH20 [27], by in situ hybridization. Other researchers carried out more extensive expression mappings in mouse, for example, Honjo et al. [28] in the postnatal retina (CDH2, CDH4, CDH6, CDH8 and CDH11) and Xu et al. [29] throughout retinal development (CDH1, CDH2 and CDH3). Each of these studies revealed that cadherins are expressed in a temporally and spatially restricted fashion in the retina, as shown previously for the brain (see above). However, a comprehensive study of the expression of multiple cadherins throughout all stages of retinal development in a single mammalian species is missing to date, especially for δ-protocadherins.

**Figure 1:**
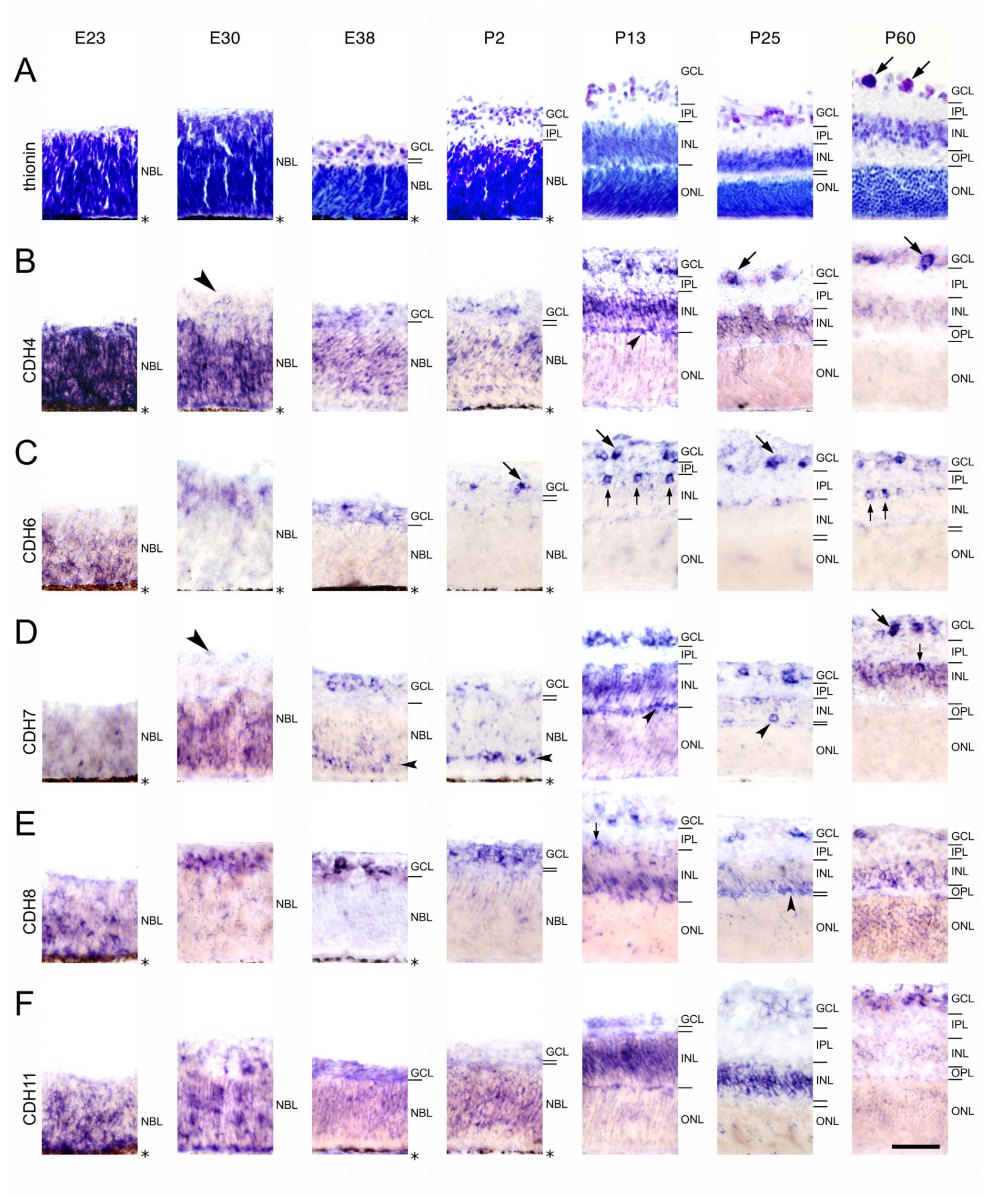
**Histology and differential expression of cadherins in central regions of the developing ferret retina**. Expression of cadherin-4 (CDH4; B), cadherin-6 (CDH6; C), cadherin-7 (CDH7; D), cadherin-8 (CDH8; E) and cadherin-11 (CDH11; F) was mapped with cRNA probes at embryonic day 23 (E23), E30, E38, postnatal day 2 (P2), P13, P25, and at the mature stage (P60). For neuroanatomical orientation, Nissl-stained sections (thionin) are shown in A. The different layers of the retina are indicated on the right side of each panel. Large arrows point at large ganglion cells (> 15 μm in largest diameter) in the ganglion cell layer (GCL). Small arrows point at single, large cells of the inner nuclear layer (INL) adjacent to the inner plexiform layer (IPL). Large arrowheads point at negative inner layers of the neuroblast layer (NBL). Small arrowheads point at putative horizontal cells adjacent to the outer plexiform layer (OPL) or putative developing horizontal cells in the NBL, respectively. The outer nuclear layer (ONL) is shown up to the outer limiting membrane and does not include the outer photoreceptor segments after P13. The pigment epithelium next to the neuroblast layer (NBL) is indicated by an asterisk. Scale bar in F, 50 μm, applies to all panels.

The ferret visual system serves as a suitable experimental model for visual development; it shows close similarity to the cat visual system [30,31]. Compared to the cat, the shorter gestational period of 42 days and a larger portion of postnatal development are of great advantage. Greiner and Weidman [32,33] described ferret retinogenesis in detail (see nuclear staining in Fig. 1A). 23 days after conception (E23), the ferret neural retina consists of a pseudostratified neuroepithelium, the neuroblast layer (NBL). Ganglion cells are the first retinal neurons that become postmitotic and migrate towards the vitreous side. At E38, the ganglion cell layer (GCL) has completely separated from the NBL, creating the inner plexiform layer (IPL) in between the other two layers. Most horizontal cells and cone photoreceptors become postmitotic and migrate to their final position before birth; most amacrine cells are differentiated around birth [for rat, see [34]]. In the second postnatal week, the NBL divides into an outer nuclear layer of photoreceptors (ONL) and an inner nuclear layer of interneurons (INL), with the outer plexiform layer (OPL) in between. All structural parts of the retina have formed at the time of eye opening, which is not until 30 days after birth. At P60, only the photoreceptor outer segments and the INL have changed their size, and the histology of the ferret retina can be considered mature. Note that the thickness of the retina does not increase linearly throughout development but also decreases intermittently due to stretching of the retina when the diameter of the eye bulb is growing [Fig. 1A; see also [32]].

In the present study, we investigate the developmental expression of seven classic cadherins (CDH4, CDH6, CDH7, CDH8, CDH11, CDH14, CDH20) and eight δ-protocadherins (PCDH1, PCDH7, PCDH8, PCDH9, PCDH10, PCDH11, PCDH17, PCDH18) in a single species, the ferret, by in situ hybridization. Finally, we compare our results with previous data about cadherin expression in the retina.

## Results and Discussion

### General Results

**Figure 2:**
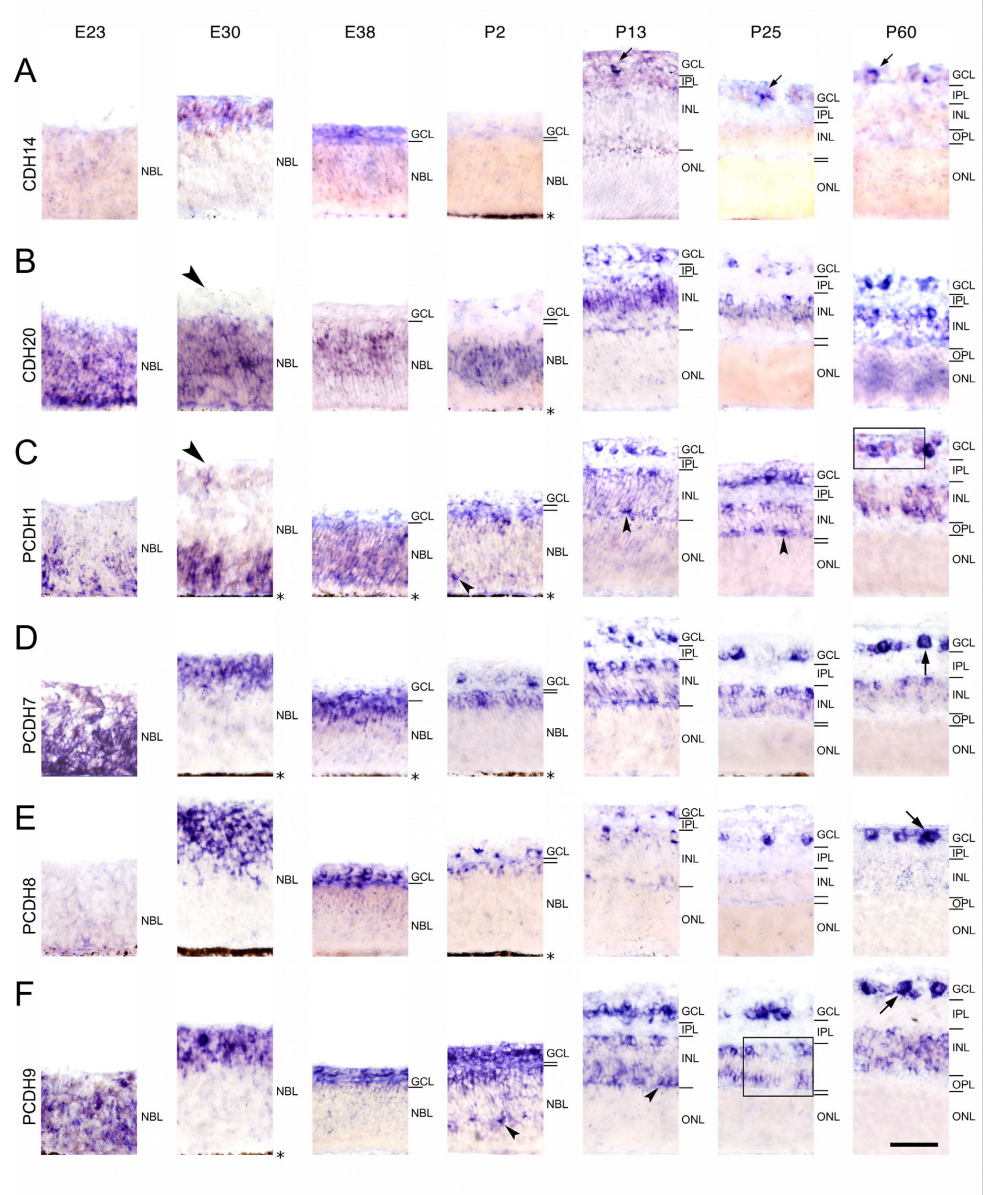
**Differential expression of cadherins in central regions of the ferret retina during development**. Expression of cadherin-14 (CDH14; A), cadherin-20 (CDH20; B), protocadherin-1 (PCDH1; C), protocadherin-7 (PCDH7; D), protocadherin-8 (PCDH11; E) and protocadherin-9 (PCDH9; F) was mapped with cRNA probes at embryonic day 23 (E23), E30, E38, postnatal day 2 (P2), P13, P25 and at the mature stage (P60). For neuroanatomical orientation, the different layers of the retina are indicated on the right side of each panel. Large arrows point at large ganglion cells (> 15 μm in largest diameter), whereas small arrows point at several cells of the ganglion cell layer (GCL) in A (CDH14). Large arrowheads point at negative inner layers of the neuroblast layer (NBL). Small arrowheads point at putative horizontal cells adjacent to the outer plexiform layer (OPL) or putative developing horizontal cells in the NBL, respectively. The outer nuclear layer (ONL) is shown up to the outer limiting membrane and does not include the outer photoreceptor segments after P13. The pigment epithelium next to the NBL is indicated by an asterisk. Areas boxed in C and F are shown at a higher magnification in Figure 4B and G, respectively. Other abbreviations: INL, inner nuclear layer; IPL, inner plexiform layer. Scale bar in F, 50 μm, applies to all panels.

**Figure 3:**
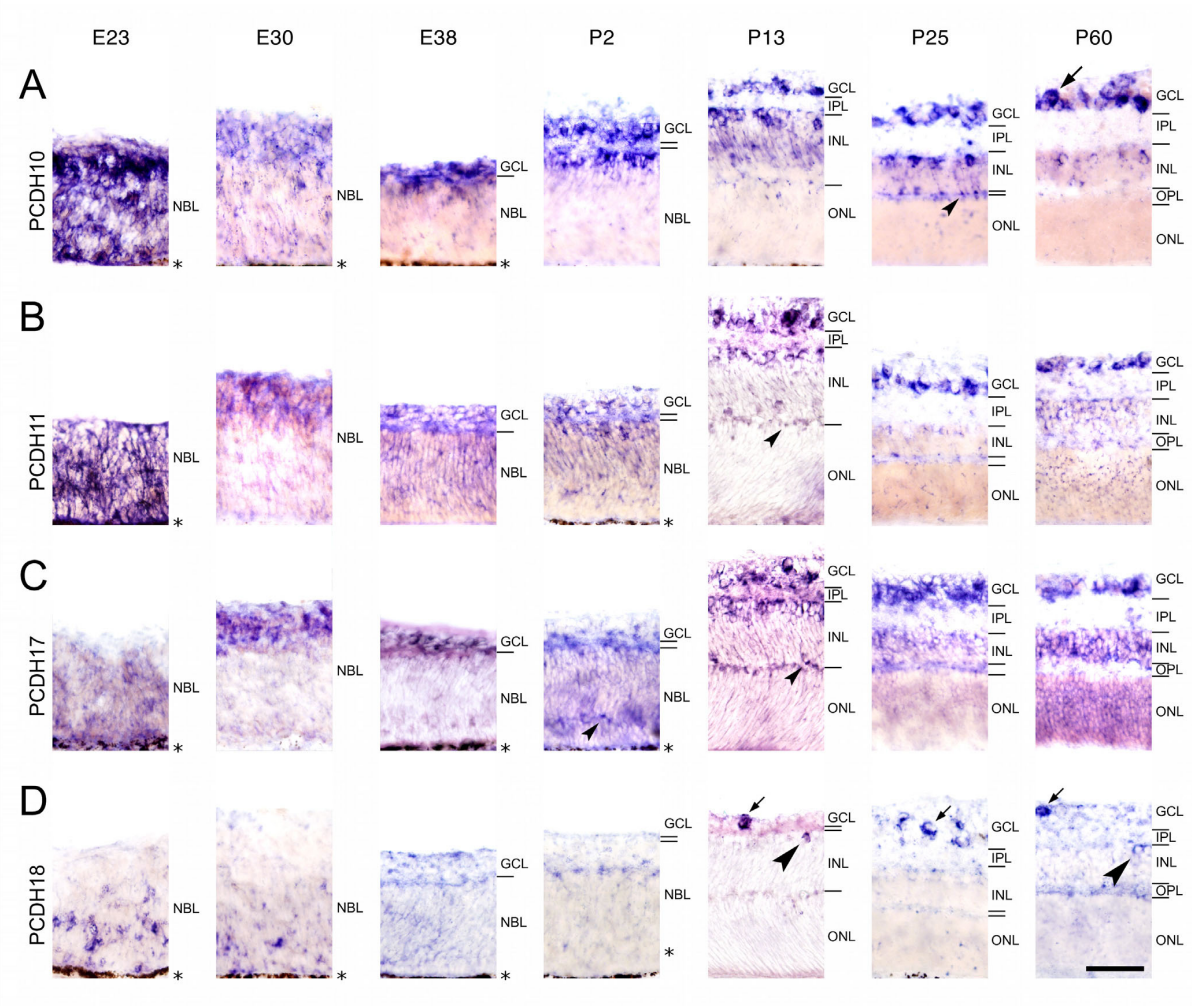
**Differential expression of cadherins in central regions of the ferret retina during development**. Expression of protocadherin-10 (PCDH10; A), protocadherin-11 (PCDH11; B), protocadherin-17 (PCDH17; C) and protocadherin-18 (PCDH18; D) was mapped with cRNA probes at embryonic day 23 (E23), E30, E38, postnatal day 2 (P2), P13, P25 and at the mature stage (P60). For neuroanatomical orientation, the different layers of the retina are indicated on the right side of each panel. Large arrows point at large ganglion cells (> 15 μm in largest diameter), whereas small arrows point at several cells in the ganglion cell layer (GCL) in D (PCDH18). Large arrowheads point at amacrine cells at the inner margin of the inner nuclear layer (INL), next to the inner plexiform layer (IPL). Small arrowheads point at putative horizontal cells adjacent to the outer plexiform layer (OPL). The outer nuclear layer (ONL) is shown up to the outer limiting membrane and does not include the outer photoreceptor segments after P13. The pigment epithelium next to the neuroblast layer (NBL) is indicated by an asterisk. Scale bar in F, 50 μm, applies to all panels.

All 15 cadherins examined are expressed in the developing neural retina of the ferret in different locations, continuously from early embryonic to adult stages (Figs. 1, 2, 3). Exceptions are CDH14 and PCDH18, which are expressed very weakly and with interruptions. All cadherins exhibit a restricted, spatiotemporally regulated expression pattern. Each cadherin is expressed in specific layers, sublayers and subsets of cells at different stages (see Table 1). Thus, each retinal layer is marked by the combinatorial expression of multiple classic and δ-protocadherins during development (see Table 2). In general, cadherin expression is especially widespread and intense at P13. After P13, expression patterns remain relatively stable, although gradual changes are observed. Sense probes, which served as a control, did not result in any significant signal (data not shown). The pigmented epithelium is not visible in all panels of Figures 1, 2, 3 because, in some cases, albino animals were used or retinal detachment occurred. Images from P13 and later stages show the neural retina without the photoreceptor outer segments, which are devoid of in situ signal. Peripheral regions of the retina show expression patterns similar to central ones (data not shown). Nevertheless, a maturational delay of around 10 to 15 days has been reported for extreme peripheral parts of the ferret retina [35]. Dorsoventral differences of ganglion cell distribution have also been reported [36]. For identification of retinal layers and cell types, we mainly refer to the histological study of the developing ferret retina by Greiner and Weidman [32,33], to some detailed morphology studies [31,37,38] and to previous cadherin expression studies in the developing vertebrate retina (see Introduction).

**Table 1:** Cadherin expression in the developing ferret retina.

**CDH4**	NBL	E23: (a)+++; E30: (a)+++, except inner tier; E38, P2: (n)++	
	GCL	E38—P60: (n)++, large ganglion cells +++	
	INL	inner	P13: (n)++; P25: (s)+; P60: -
		middle	P13: (a)+++; P25: (a)++; P60: (n)+
		outer	P13: patchy signal; P25, P60: -
	ONL	-	
			
**CDH6**	NBL	E23: (n)++; E30: (n)+, only inner layers; E38, P2: (s)+, only inner cells	
	GCL	E38—P60: (s)++, mainly large ganglion cells +++	
	INL	inner	P13—P60: (n)++, single larger cells +++
		middle	P13: (s)+; P25—P60: -
		outer	P13—P60: -
	ONL	-	
			
**CDH7**	NBL	E23: (n)+; E30: (n)++, only outer two thirds; E38, P2: (n)+, one outer layer (n)+++	
	GCL	E38—P60: (n)++, large ganglion cells +++	
	INL	inner	P13: (n)++; P25: (n)+; P60: (n)++; single larger cells +++
		middle	P13: (a)+++; P25: (s)+; P60: (n)++, only inner part
		outer	P13: (n)++; P25: (s)++; P60: -
	ONL	P13: (n)+; P25—P60: -	
			
**CDH8**	NBL	E23: (a)++; E30: (n)++, only inner layers; E38, P2: -	
	GCL	E38, P2: (n)++; P13—P60: (s)+	
	INL	inner	P13: (s)++; P25, P60: -
		middle	P13: (n)++, only outer part; P25: (n)+ P60: (n)++
		outer	P13: (n)++; P25, P60: (n)+
	ONL	P13, P25: -; P60: (a)+	
			
**CDH11**	NBL	E23, E30: (a)+++; E38, P2: (a)++, outermost layer -	
	GCL	E38—P25: (n)+; P60: (n)++	
	INL	inner	P13: (n)+; P25, P60: -
		middle	P13, P25: (a)+++; P60: (s)+
		outer	P13—P60: -
	ONL	-	
			
**CDH14**	NBL	E23: -; E30: (n)+, only innermost layers; E38: (n)+; P2: -	
	GCL	E38: (n)+; P2: -; P13—P60: (s)+	
	INL	inner	P13: (s)+; P25, P60: -
		middle	P13—P60: -
		outer	P13—P60: -
	ONL	-	
			
**CDH20**	NBL	E23: (a)+++; E30: (a)++, except inner tier; E38, P2: (n)++, only middle layers	
	GCL	E38, P2: -; P13—P60: (n)++	
	INL	inner	P13, P25: (s)++; P60: (n)++
		middle	P13: (a)+++; P25: (n)++; P60: (n)++
		outer	P13—P60: -
	ONL	P13, P25: -; P60: (n)+	
			
**PCDH1**	NBL	E23, E30: (n)++, inner two thirds (s)+; E38: (a)++, outermost layer -; P2: (n)++, (s)+++	
	GCL	E38, P2: (s)+; P13—P60: (n)+++	
	INL	inner	P13—P60: (n)++, single larger cells ++
		middle	P13: (n)+; P25, P60: (s)+
		outer	P13—P60 (n)++
	ONL	-	
			
**PCDH7**	NBL	E23: (a)+++; E30- P2: (n)+++, only inner layers	
	GCL	E38: (n)++, only outer cells; P2: (s)++; P13—P60: (n)++, most large ganglion cells +++	
	INL	inner	P13—P60: (n)+++, single larger cells ++
		middle	P13, P25: (n)+; P60: (s)+
		outer	P13, P25: (n)++; P60: -
	ONL	-	
			
**PCDH8**	NBL	E23: -; E30: (n)++, only inner half; E38, P2: (n)+, only innermost cells	
	GCL	E38: (n)+++; P2: (n)++; P13, P25: (s)++; P60: (n)++, large ganglion cells +++	
	INL	inner	P13: (n)++; P25, P60: -
		middle	P13—P60: -
		outer	P13—P60: -
	ONL	-	
			
**PCDH9**	NBL	E23: (a)+++; E30, E38: (n)++, only inner layers; P2: (n)++, one outer layer (s)+++	
	GCL	E38-P60: (a)++, large ganglion cells +++	
	INL	inner	P13-P60: (n)++, single larger cells ++
		middle	P13: (n)+; P25: -; P60: (n)++
		outer	P13: (a)++; P25: (n)++; P60: (n)++
	ONL	-	
			
**PCDH10**	NBL	E23: (a)+++; E30: (n)++; E38, P2: (n)++, only inner layers	
	GCL	E38, P2: (a)+++; P13—P60: (n)++, large ganglion cells +++	
	INL	inner	P13—P25: (n)++; P60: (n)+; single larger cells ++
		middle	P13: (n)++; P25: (n)+; P60: -
		outer	P13: patchy signal; P25: (s)++; P60: -
	ONL	-	
			
**PCDH11**	NBL	E23: (a)+++; E30: (n)+, only inner layers; E38, P2: (n)++	
	GCL	E38—P60: (n)++	
	INL	inner	P13: (n)++; P25: (s)+; P60: (n)+
		middle	P13: (n)+; P25: -; P60: (n)+
		outer	P13—P60: (s)+
	ONL	P13, P25: -; P60: (a)+	
			
**PCDH17**	NBL	E23: (n)+; E30: (n)++, only inner layers; E38: -; P2: (a)+, one outer layer (n)++	
	GCL	E38—P60: (n)++	
	INL	inner	P13: (a)++; P25, P60: (n)++
		middle	P13—P60: (a)+
		outer	P13—P60: (n)+
	ONL	P13—P60: (a)+	
			
**PCDH18**	NBL	E23, E30: (s)++; E38, P2: -	
	GCL	E38, P2: -; P13—P60: (s)++	
	INL	inner	P13—P60: (s)+
		middle	P13—P60: -
		outer	P13—P60: -
	ONL	-	

Approximate number of positive cells: -, no cell positive; (s), single cells (up about 20%) positive; (n), numerous cells (about 20—80%) positive; (a), all or almost all cells (more than 80%) positive. Expression level: -, absent; +, weak; ++, moderate; +++, strong.

**Table 2:** Summary of cadherin expression in the retinal layers.

	NBL	GCL	inner INL	middle INL	outer INL	ONL
CDH4	+++	++	++	+++	+	-

CDH6	++	+	++	+	-	-

CDH7	++	++	++	+++	++	(+)

CDH8	+++	+	+	++	++	-

CDH11	+++	++	-	+++	-	-

CDH14	-	+	(+)	-	-	-

CDH20	+++	++	+	+++	-	-

PCDH1	++	++	++	+	++	-

PCDH7	+++	++	++	++	++	-

PCDH8	-	+	+	-	-	-

PCDH9	+++	+++	++	++	+++	-

PCDH10	+++	++	++	++	+	-

PCDH11	+++	++	++	++	+	-

PCDH17	++	++	+++	+++	++	(+)

PCDH18	+	+	+	-	-	-

Expression level was roughly determined at E23 (NBL) and P13 (all other layers). Abbreviations: -, no cell positive; +, single cells (up about 20%) positive; ++, numerous cells (about 20-80%) positive; +++, all or almost all cells (more than 80%) positive; (+) faint expression

#### Neuroblast layer (NBL)

At E23, a strong expression of some cadherins marks the entire neuroepithelium, whereas other cadherins mark only a subpopulation of NBL cells or start expression later, respectively. Remarkably, the outer, proliferative zone of the NBL is selectively marked by CDH4, CDH7 and CDH20 at E30 (arrows in Figs. 1B, 1D, 2B). PCDH1 expression is restricted to the outermost layers (Fig. 2C, E30). In contrast, most cadherins exclusively label the innermost retinal zone, which contains prospective ganglion cells and amacrine cells, before birth. These two cell types display the same cadherin expression profile, respectively, also at later stages of development (for example, see Fig. 2E). Remarkably, in the early retina, the cadherin-expressing cell populations do not show borders that run strictly in parallel to the retinal surface but form cluster-like protrusions into adjacent zones (for example, CDH4 or CDH7 at E30 in Fig. 1).

#### Ganglion cell layer (GCL)

**Figure 4:**
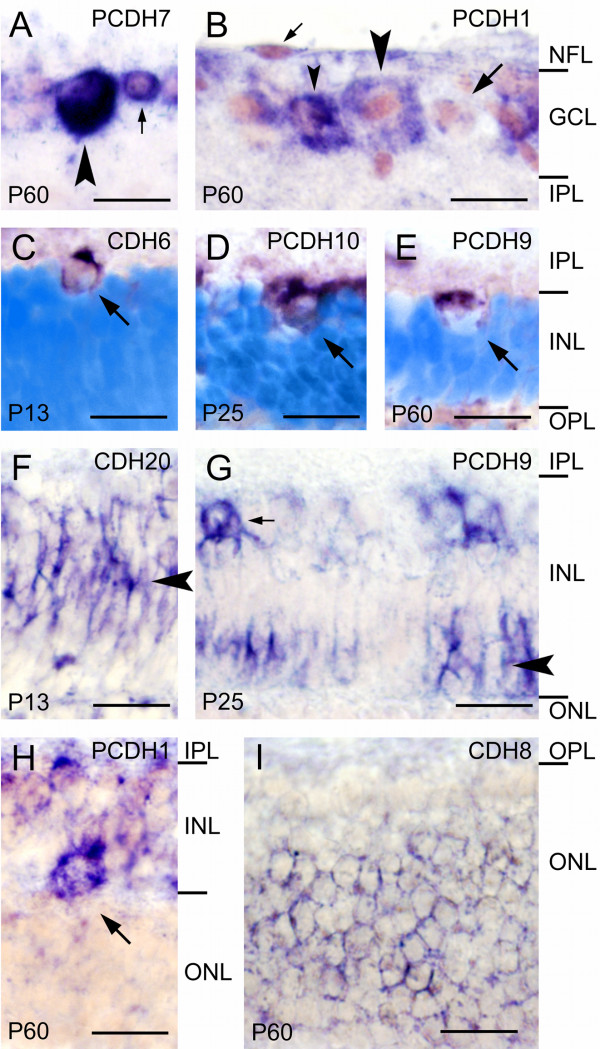
**Cell type-specific expression of cadherins revealed by in situ hybridization (dark purple precipitate)**. A, B: Expression of protocadherin-7 (PCDH7; A) and protocadherin-1 (PCDH1; B) in the ganglion cell layer (GCL) at postnatal day 60 (P60). Note the positive large ganglion cells (large arrowhead in A, 19 μm diameter; large arrowhead in B, 26 μm; small arrowhead in B, 20 μm), a negative large ganglion cell (large arrow in B, 25 μm), and a positive, putative small ganglion cell (small arrow in A, 11 μm). The elongated cell in the nerve fiber layer (NFL, small arrow in B) probably represents an astrocyte. Panel B shows the area boxed in Fig. 2C. C-E: Putative displaced ganglion cells adjacent to the inner plexiform layer (IPL; arrows) express cadherin-6 at P13 (CDH6; C), protocadherin-10 at P25 (PCDH10; D), and protocadherin-9 at P60 (PCDH9; E). Cells are identified as ganglion cells based on their large somata (13 μm, 15 μm, and 14 μm, respectively) and nuclei (10 μm, 11 μm, and 12 μm, respectively) and the large gaps occupied by them between neighboring amacrine cells. F, G: Cadherin-20 expression by putative Müller glia at P13 (CDH20, arrowhead in F) and PCDH9 expression by putative bipolar cells (arrowhead in G). The small arrow marks a positive amacrine cell. Panel G shows the area boxed in Fig. 2F. H: A large horizontal cell expressing protocadherin-1 at P60 (PCDH1; arrow). I: Photoreceptor cells throughout most of the outer nuclear layer (ONL) express cadherin-8 at P60 (CDH8; arrowhead). Cell nuclei are shown in light pink color (A, B, F-I) or light blue color (Hoechst nuclear counterstain; C-E). The different layers of the retina are indicated on the right-hand side of each row of panels. Other abbreviations: INL, inner nuclear layer; OPL, outer plexiform layer. Scale bars are 20 μm.

The ganglion cell layer shows the most prevalent and consistent pattern of expression for all cadherins up to adulthood. According to Vitek et al. and Henderson et al. [31,36], the cells with diameters of more than 15 μm in the postnatal GCL include mainly ganglion cells ("large" ganglion cells). The largest ganglion cells in our sample had a soma of 26 μm in diameter. The majority of cells in the GCL has a diameter of less than 15 μm and comprise other ganglion cell types as well as numerous inverted amacrine cells and glia, which are not distinguishable by our methods ("small cells of the GCL"). Apparently all cadherins label large ganglion cells or a subset thereof (for example, see large arrows in Figs. 1, 2 &3, and arrowheads Figs. 4A and 4B).

Astrocytes are rarely seen (see small arrow in Fig. 4B). The study of these non-neuronal cells as well as blood vessel cells and microglia is beyond the scope of the present investigation.

#### Inner nuclear layer (INL)

Most cadherins are expressed by amacrine cells from E38 onward, the earliest stage, at which developing amacrine cells can be identified on the basis of their location. After P13, when most amacrine cells have differentiated [[32], for rat, see [34]], they stop expression of some cadherins (CDH4, CDH8, CDH11, PCDH8) while they begin to express CDH20 at P13; CDH14 faintly labels amacrine cells only around P13.

A few large positive cells are sparsely scattered along the inner margin of the INL (for example, see Fig. 4C-E). Cells of similar morphology have been described as displaced ganglion cells in other species [for chicken, see [24,39]]. Even if the size of their soma and nucleus is similar to large amacrine cells around P13 (on average, 12.9 μm ± 0.2 SD and 9.8 μm ± 0.5, respectively), they can be distinguished from amacrine cells by their sparse distribution, their occupation of large gaps between neighboring cells as well as by their relatively large somata and nuclei after P13 up to adulthood (on average, 14.6 μm ± 0.9 and 11.9 μm ± 0.7, respectively, at P60). Displaced ganglion cells apparently express CDH7, PCDH1, PCDH7 (data not shown), PCDH9 (Fig. 4E) and, most prevalent, CDH6 (Fig. 4C) and PCDH10 (Fig. 4D) at several stages. Nevertheless, our morphological analysis could have missed smaller displaced ganglion cells.

Noteworthy, CDH6 strongly labels numerous large cell bodies at the inner margin of the INL from P13 onward (small arrows in Fig. 1C, P13 and P60). These cells are smaller than large ganglion cells (12.7 ± 0.8 μm [mean ± SD] at P13, and 13.0 ± 0.5 μm at P60), have a relatively small nucleus (not more than 9 μm) and are sometimes spaced not more than 30 μm apart (Fig. 1C, P13 and P60). Because they retain a medium size also at later stages, are numerous and densely packed in between similar but smaller cells, these cells may represent a distinct type of large amacrine cells rather than displaced ganglion cells.

The middle part of the inner nuclear layer, which primarily contains the cell bodies of Müller glial cells and some inner bipolar cells, is positive for CDH4, CDH7, CDH11, CDH20 and PCDH10 (for example, see arrowhead in Fig. 4F). The outer part of the INL, which mainly contains bipolar cell somata, expresses CDH8, PCDH7 and PCDH9 (for example, see arrowhead in Fig. 4G).

Some single cells express PCDH1, PCDH9 and PCDH17 in a sublayer of the outer NBL from P2 onward (small arrowheads in Figs. 2C, F; 3C). Already at E38, CDH7 is expressed by some cells at this location (small arrowhead in Fig. 1D, E38). This sublayer anticipates the later splitting of the NBL (small arrowheads in Fig. 1D, P2 and P13). According to their radial position and distribution, we tentatively identify them as horizontal cells. At later stages, cadherin-expressing horizontal cells can be more clearly identified morphologically (for example, see small arrowhead in Fig. 1D, P25, and Fig. 4H). Around P13, some patchy signal is observed transiently at the outer margin of the INL for some cadherins (CDH4, CDH8, PCDH10, PCDH11; see small arrowheads in Figs. 1B, P13; 1E, P25; 3A, P25; 3B, P13); it is possible that these signals are also associated with horizontal cells or their proximal processes. Interestingly, these mRNA signals disappear from most of this site by the time of eye opening when horizontal cells have differentiated.

#### Outer nuclear layer (ONL)

The ONL is not visible with most cadherin probes. Transiently, the ONL expresses a few cadherins faintly around P13 (CDH7 and PCDH17), when the photoreceptor outer segments grow out, as well as at the mature stage (CDH8, CDH20, PCDH11, PCDH17; for example, see arrowhead in Fig. 4I). Prospective photoreceptor cells do not express most of the cadherins at E38 and P2 in the outermost layers of the NBL.

### General Discussion

To our knowledge, this study is the first to map the developmental expression of CDH14, PCDH1, PCDH7, PCDH8, PCDH9, PCDH11, PCDH17 and PCDH18 during development of the vertebrate retina. Moreover, for the first time, the expression of cadherins was mapped in the retina of the ferret. In contrast to previous studies in other vertebrate species [24,25,28,29,40,41] multiple classic cadherins and δ-protocadherins were mapped in detail throughout retinogenesis.

Each of the cadherins studied exhibits a unique expression pattern, although some similarity and overlap between cadherins are observed. In general, until P13, expression is restricted to proliferating and differentiating cell populations. This observation is in agreement with the morphogenetic roles of cadherins in the CNS and vertebrate retina (see Background). Changes in cadherin expression patterns coincide with specific steps of neuronal development; in some cases, developmental events are even anticipated before they become visible in Nissl or nuclear stainings. This anticipation is especially evident around E30 and from P2 to P13, when major advances in retinal development occur. For example, the innermost layers of the NBL, which mainly include prospective ganglion cells, express selectively particular cadherins at E30. The same cadherins mark ganglion cells in the later GCL (for example, see Figs. 1E, 3C) and sometimes, in addition, amacrine cells in the later INL (for example, see Figs. 2D, 2E and 2F). Vice versa, CDH20 does not label the innermost layers of NBL at E30 (see arrow in Fig. 2B, E30) and the GCL is also negative for this molecule at later stages. Moreover, signal in some outer layers consistently marks the radial position of the future INL, especially its later positive middle part, where Müller cells reside. Strikingly, putative developing horizontal cells are marked by some cadherins from E38 onwards (for example, see small arrowheads in Fig. 1D). Although the identification of cell types and subtypes required confirmation by double labeling with cell type-specific markers, the anticipation of histological transformations by cadherin expression seems evident on histological grounds.

As development proceeds, specific combinations of cadherins turn into markers for different retinal cell types or subpopulations thereof. Because each cell type resides in a particular retinal layer, most cadherins exhibit a layer-specific expression profile at later stages of development. Thus, each retinal layer is marked by the combinatorial expression of multiple cadherins (see Table 2). A layer-specific expression of cadherins has been described previously for the germinal zones and layers of the cerebral cortex [42]. We did not find that any of the cadherins studied by us was expressed by only a single retinal cell type. Cadherins with this type of highly restricted expression are the evolutionary ancient horizontal cell cadherin in nonmammalian animals [cHZ-cadherin; [43]] and the photoreceptor-specific PCDH21 [prCAD; [44]],

The cadherins studied continue to be expressed at high levels after neurogenesis, during the period of neurite outgrowth and neural circuit formation. Most strikingly, ganglion cells, especially large ones, express almost all cadherins at high levels from P13 onward. Many of the cadherins examined are expressed among neuronal populations that are synaptically connected to each other, like amacrine cells and ganglion cells. Moreover, expression profiles seem to be highest in these populations during the peak of intraretinal circuit formation (from P13 onward), whereas mRNA signal begins to decrease in other populations at this stage of development. It remains to be studied at the protein level whether arborization and synaptogenesis depend on the homotypic binding of simultaneously expressed cadherin molecules or on a temporally restricted competence of ingrowing axons to interact with multiple adhesion molecules already present in the area, as Petrovic and Hummel [45] proposed. Alternatively, Prakash et al. [46] suggested a "two step process" including rough cadherin-dependent target recognition followed by a more specific cadherin-independent synaptogenesis. The persistence of cadherin expression into adulthood in subsets of cells suggests that cadherins may also help to sustain the proper function of the mature neural retina.

In summary, our findings suggest that cadherins may provide a combinatorial adhesive code that specifies developing retinal cell populations and neural circuits during retinogenesis and in the mature retina [9]. This adhesive code may be based on qualitative as well as quantitative differences in adhesiveness between retinal cells expressing multiple cadherins and other adhesive molecules [28]. The functional role of cadherins in brain development has been studied extensively in the retina and other parts of the CNS (see Background). To relate these functional studies to the present expression data is beyond the scope of this paper.

### Comparison between Ferret, Mouse and Chicken

Our results confirm previous studies in other vertebrates [24,25,28,29,40,41], showing that cadherins are subject to a tight temporal and spatial regulation during retinogenesis. In particular, compared to the previously published expression patterns for chicken and mouse retina, we generally found similar expression in developing ferret for most of the cadherins. However, we also noted the following differences.

The prominent expression of PCDH10 mRNA in the GCL sets in significantly later in chicken [25] than in ferret. In the ferret, mRNAs for CDH4, CDH6 and CDH7 are found in the NBL as early as E23 and E30. At comparable stages in the chicken (E5-E8), the same cadherins cannot be detected in the NBL by immunostaining [24,41]. Because a temporal delay between mRNA and protein expression is rarely observed for cadherins in the developing vertebrate nervous system [reviewed in [17]], it is more likely that the expression of the cadherins is differentially regulated in the retina of avian species and mammals. This notion is supported by the finding that CDH4 is intensively expressed by ganglion cells in ferret (this study) and mouse [28] but not in chicken [24,41]. Additionally, an expression of CDH6 is observed at the outer margin of the INL in chicken [24] but neither in mouse [28] nor in ferret.

An example for differences between ferret and mouse is observed in the INL where CDH4 is expressed in single horizontal and amacrine cells in both the mouse [28] and the ferret, but in the ferret, CDH4 is expressed additionally in the outer zone of Müller cells and bipolar cells. Another example is the expression of CDH8 and CDH11 in a subset of ganglion cells that is prominent in both species at intermediate stages of development; at late stages, however, CDH8 and CDH11 expression by GCL cells is seen in ferret but not in mouse [28]. Moreover, although the early embryonic expression of CDH20 is similar in mouse and ferret, this molecule is prominently expressed in the GCL already at embryonic mouse stages [40] while the expression in ferret sets in postnatally at P13.

The observed differences are minor and may relate to species differences in the genetic regulation of retinal morphogenesis. Similar species differences have been described for other morphoregulatory factors [reviewed in [6]]. Overall, the observed general similarities in cadherin expression suggest evolutionarily conserved functions of cadherins in retinal development.

## Conclusions

Fifteen classic cadherins and δ-protocadherins were found to be expressed differentially by restricted populations of retinal cells during retinogenesis in the ferret. The observed expression patterns anticipate histogenetic changes that become visible in nuclear or Nissl stainings at later stages. A comparison to previous results in chicken and mouse reveals overall conserved expression patterns of some cadherins but also species differences. The remarkable spatiotemporally restricted fashion of cadherin expression in the developing ferret retina suggests that cadherins provide a combinatorial adhesive code that might specify developing retinal cell populations and intraretinal as well as retinofugal neural circuits during retinogenesis. A more detailed classification of the different cadherin-positive retinal cell classes would require double-labeling with cell type-specific antibodies at the protein level or lineage analysis in transgenic animal models. The expression patterns described in the present work provide a basis for experiments that investigate cadherin-related morphogenetic events during mammalian retinal development.

## Methods

### Animals and Preparation of Tissues

Ferrets (*Mustela putorius furo*) bred in captivity were purchased from the Federal Institute of Risk Assessment (Berlin-Marienfelde, Germany). Under deep anesthesia by an overdose of intraperitoneal pentobarbital, animals used in this study were quickly decapitated. National and institutional guidelines on the welfare of animals in research were strictly followed. We studied eyes of embryos delivered by cesarean section from timed pregnant ferrets at 23, 30 and 38 days after conception (E23, E30, E38) and postnatal eyes of 2, 13, 25 and 60 days old animals (P2, P13, P25, P60). These stages represent major steps in retinal development. Day of birth was designated as P0. The number of animals used in this study was kept to a minimum necessary to produce reliable data. Efforts were made to minimize animal suffering.

For E23 and E30 embryos, eyes were left in the skull and fixed in situ. From E38 onward, eyes were enucleated for fixation. From P2 onward, the lens was removed from the eye to allow for better penetration of the fixative. Specimens were immersed overnight in ice-cold 4% formaldehyde dissolved in phosphate-buffered salt solution (PBS; 13 mM NaCl, 7 mM Na_2_HPO_4_, 3 mM NaH_2 _PO_4_; pH 7.4). After incubation in a graded series of sucrose solutions (12%, 15% and 18%) for cryoprotection, specimen were embedded in Tissue Tek (Science Services, Munich, Germany), frozen in liquid N_2 _and stored at -80°C.

Consecutive series of 20 μm-thick sections were cut in a microtome-cryostat (HM 560 Cryo-Star, Microtome International, Walldorf, Germany). They were directly thawed onto SuperFrost Plus slide glasses (Menzel, Braunschweig, Germany) and dried at 50°C. In total, sections through the eyes were cut from 3 whole embryos (E23), 3 embryonic heads (E30) and 14 enucleated eyes (E38 to P60, 2-4 eyes per stage).

### cRNA Probe Synthesis

Digoxigenin-labeled antisense cRNA probes were synthesized for 7 classic cadherins and 8 δ-protocadherins, as described by Krishna et al. [42]. The complementary DNA fragments of 1.1 to 3 kb length used for probe synthesis are listed in Table 3. After linearized plasmids were transcribed with T7 or SP6 RNA polymerase (New England Biolabs, Ipswich, MA), we used the DIG RNA Labeling Kit (Roche Diagnostics, Mannheim, Germany) to generate digoxigenin-labeled sense and antisense probes. Probes were purified by LiCl/EtOH precipitation or by using Quick Spin Columns (Roche Diagnostics). Probe size was verified by formaldehyde agarose gel electrophoresis.

**Table 3:** Size and accession number of the cadherin fragments used for in situ hybridization.

Cadherin	Size (bp)	GenBank number
CDH4	1501	EU665238
CDH6	1824	EU665239
CDH7	1824	EU665240
CDH8	1825	EU665241
CDH11	1110	EU665242
CDH14	1474	EU665243
CDH20	2000	EU665244
PCDH1	1961	EU665245
PCDH7	2326	EU665246
PCDH8	1702	EU665247
PCDH9	1749	EU665248
PCDH10	1860	EU665249
PCDH11	2044	EU665250
PCDH17	2038	EU665251
PCDH18	2351	EU665252

### In Situ Hybridization

In situ hybridization was performed according to a previously published protocol [47]. In brief, we postfixed cryostat sections with 4% formaldehyde in PBS at 4°C. For better probe penetration, sections were pretreated with 1 μg/ml proteinase K (Sigma, Steinheim, Germany) in 100 mM Tris (pH 8.0), 50 mM EDTA for 5 minutes and with 0.25% acetic anhydride in triethanolamine buffer. Sense and antisense cRNA probes at a concentration of about 1.5 ng/μl in hybridization buffer (50% formamide, 10 mM EDTA, 3× SSC, 1× Denhardt's solution, 10× dextran sulfate, 42 μg/ml yeast RNA, 42 μg/ml salmon sperm DNA) were applied to the sections and hybridized overnight at 70°C in a humid chamber. After several washing steps and RNase A treatment (20 μg/ml in NTE buffer [10 mM Tris, 1 mM EDTA, 0.5 mM NaCl, pH 8.0]), alkaline phosphatase-coupled anti-digoxigenin Fab fragments (Roche Diagnostics, Mannheim, Germany) were applied overnight at 4°C. Sections were washed and reacted with a substrate mixture of nitroblue tetrazolium salt (NBT; 75 mg/ml) and 5-bromo-4-chloro-3-indolyl-phosphate (BCIP; 50 mg/ml) in alkaline buffer for 1-3 days at 4°C or at room temperature, until enough reaction product had formed. Sections were mounted in Entellan (Merck, Whitehouse Station, New Jersey). For nuclear counterstaining, some sections were stained with Hoechst 33342 (Sigma) in PBS and mounted in Mowiol (Roche Molecular Biochemicals, Mannheim, Germany). The in situ hybridization procedure was performed up to 5 times for each probe and stage to verify the results.

All sections were examined under a light transmission/fluorescence microscope (BX40, Olympus, Hamburg, Germany) equipped with a digital camera (DP70, Olympus, Hamburg, Germany). Finally, selected areas of interest were cropped from the photomicrographs and adjusted in contrast, brightness and tonal value with the gimp software (GNU Image Manipulation Program, under auspices of the GNOME project) and Photoshop software (Adobe Systems, Mountain View, CA, USA) for optimal display of staining patterns. Overlays of nuclear counterstaining and in situ data were generated.

Retinal cell types were identified on the basis of their radial position, morphology and embedding between neighboring cells. For determination of cell soma size of large CDH6-positive amacrine cells (see below), the largest diameter of 10 of these cells was measured at P13 and at P60, respectively. Cell soma as well as nucleus size of 5 displaced ganglion cells positive for several cadherins were measured at P13 and P60, respectively.

## Authors' contributions

JE carried out the experiments, analyzed and documented the results and wrote the first draft of the manuscript. KK participated in the design of the study, performed the cRNA probe synthesis, carried out some of the in situ hybridizations and assisted in writing the manuscript. CR designed the study, supervised the project at all stages and edited the manuscript.
